# Food Safety Practice and Its Associated Factors among Mothers in Debarq Town, Northwest Ethiopia: Community-Based Cross-Sectional Study

**DOI:** 10.1155/2019/1549131

**Published:** 2019-06-04

**Authors:** Henok Dagne, R. P. Raju, Zewudu Andualem, Tesfaye Hagos, Kidstemariam Addis

**Affiliations:** Department of Environmental and Occupational Health and Safety, Institute of Public Health, College of Medicine and Health Sciences, University of Gondar, Ethiopia

## Abstract

**Background:**

Food safety practice is an important Public Health issue to prevent or control food-borne illnesses. Evidence shows that 10 to 20% of food-borne diseases outbreaks are because of contamination by food handlers in restaurants, butcher shops, markets, etc. However, the food safety practice and associated factors among mothers who are the food handlers at their homes are not well studied and understood. This study aimed to assess food safety practice and associated factors among mothers in Debarq town.

**Methods:**

A community-based cross-sectional study was conducted during March and April, 2018. Four hundred and twenty-three study participants were included using simple random sampling techniques. A structured questionnaire was used to collect data. Multivariable binary logistic regression analysis was used to identify factors associated with food safety practice based on AOR with 95% CI and p < 0.05.

**Results:**

About 210 (49.6%) of study participants had good food safety practice. Food safety practice of mothers was significantly associated with their educational status; secondary educational status adjusted odds ratio, AOR 3.09, 95% confidence interval, CI: 1.54, 6.20; College and University education AOR 2.95, 95% CI: 1.22, 7.12; food safety knowledge AOR 2.49, 95% CI: 1.41, 4.40; and attitude towards food safety AOR 3.67, 95% CI: 2.27, 5.94.

**Conclusion:**

Half of the study subjects had a good level of self-reported food safety practice and the other half had a poor level. Educational status, food safety knowledge, and attitude towards food safety were the identified factors associated with food safety practice. Improving the knowledge and educational status of the mothers is important to enhance their food safety practices.

## 1. Background

Food safety is an important public health issue to prevent or control food-borne illnesses. In response to the increasing number of food-borne illnesses, governments all over the world are intensifying their efforts to improve food safety [1]. According to the WHO [2], contaminated food contributes to 1.5 billion cases of diarrhoea in children each year, resulting in over three million premature deaths. However, these deaths and illnesses are shared by both developed and developing nations. The Centre for Disease Control and Prevention (CDC) estimated that food-borne diseases caused approximately 76 million illnesses annually among the United States of America's 290 million residents, as well as 325,000 hospitalisations [3, 4]. The incidence of food-borne diseases is rising in developing countries, as well as in the developed world [5].

The transmission of food-borne diseases is aggravated by unsafe food handling practices of food handlers. Approximately 10 to 20% of food-borne diseases outbreaks are because of contamination by food handlers [6]. Data about food-borne diseases in African region are still scarce [7, 8]. The role of food handlers in homes, usually mothers, in ensuring food safety at the household level is well accepted but an understanding of the status of their food handling knowledge and practices is needed [9]. Various institutional based cross-sectional studies were conducted in Ethiopia [10–14]. However, household food safety practice, particularly of mothers, is rarely studied, and the associated factors are poorly understood in Ethiopia. It is very important to understand the interaction of the prevailing food safety, knowledge, and practices of food handlers in reducing food-borne outbreaks [15]. Earlier studies [16–22] have showed that knowledge of food safety is associated with food safety practice. Other studies [23–28] have indicated that food safety and hygiene knowledge may not be translated into food safety practice. Another study showed a negative correlation between knowledge and practice [29]. This may be because surface level knowledge may not contemplate for attitudinal change to the desired level and interpreted into meaningful practice. Attitude plays a significant role in food safety practice [10, 16, 30–32]. Food safety knowledge, attitude, and practice are factors playing a fundamental role in food poisoning outbreaks prevention and control [33]. Therefore, the objectives of this community-based cross-sectional study were to assess food safety practice of mothers and identify associated factors in Debarq town.

## 2. Methods

### 2.1. Study Design and Description of Study Settings

A community-based cross-sectional study was conducted during March and April 2018 in Debarq town. Debarq is located in North Gondar zone in the northern part of Ethiopia in Amhara National Regional State 831 km from Addis Ababa (the country's capital). The 2018 estimate of population in Debarq town was 24,700 in 108.11 km^2^ [34]. A pretested structured questionnaire adapted from different literature [10–13, 35] was used to collect data. The questionnaire comprised questions about the sociodemographic characteristics of the study participants, 12 questions (washing hands after touching unwrapped raw foods, before food preparation, before touching cooked foods, after handling raw meat, after toilet visit, after sneezing or blowing nose with soap and water, wash raw food items and utensils before use, clean and sanitise food contact surfaces, separately store food and chemicals, wear jewellery when serving food, wear a hat or head covering when serving food, and prepare food with whenever a wound is on the hand) about their food safety practices with a 4-scale Likert (1-always, 2-usually, 3-sometimes, and 4-never), 10 questions (yes/no) about their knowledge of food safety (potential sources of food contamination, cross-contamination, microbial contamination, transmission of food-borne disease, personal hygiene and food-borne disease, food preservation techniques, safe food handling, person-to-person food-borne disease transmission, and hand washing knowledge), and 9 attitudinal questions with 5-scale Likert (0-strongly disagree, 1-disagree, 2-neutral, 3-agree, and 4- strongly agree). Two undergraduate students studying Environmental Health trained about the contents of a data collection tool, data collection methods, and interviewing techniques for two days were involved in the data collection process. They visited residential houses in Debarq town, interviewed 423 food handling mothers, filled the questionnaire, and rechecked and corrected the data before they left each house. Two supervisors supervised the overall interview process and daily checked the completeness, quality, and consistency of the data.

### 2.2. Source Population

All mothers in Debarq town served as a source population for the current study.

### 2.3. Study Population

Simple random sampling technique was used to select study participants. These randomly selected mothers in Debarq town were a study population for the current research.

### 2.4. Inclusion and Exclusion Criteria

Four hundred and twenty-three mothers who take part in food handling operation and who have stayed at least for 6 months in the household in Debarq town were included in the study. Mothers who were seriously ill and who did not take part in food handling because of different reasons or absent during data collection were excluded from the study.

### 2.5. Sample Size Determination and Sampling Procedure

Single population proportion formula was used to determine the sample size with the assumptions that the probability of food safety practice among mothers p = 50% because there were no other similar previous studies in Ethiopia, 95% confidence interval (CI), and 5% margin of error (d).

(1) nza/22×p×1−pd2=1.962×0.5×1−0.50.052=384

By adding 10% nonresponse rate the sample size comprises 423 mothers who participated in the study. Simple random sampling technique was used to select those study participants.

### 2.6. Data Management and Statistical Analysis

Data were entered using EPI-INFO version 7 and exported into SPSS version 20 for further analysis. For most variables, data were presented by frequencies and percentages. The variables found having a p-value less than 0.2 in the bivariable analysis were further analysed by multivariable logistic regression for controlling the effect of confounders. The variables that had a significant association with food safety practice of mothers were identified based on adjusted odds ratio (AOR) with 95% confidence interval (CI) and p < 0.05.

### 2.7. Operational Definitions

#### 2.7.1. Food Safety Practice Level

The respondents who scored less than or equal to the mean value of their responses to 12 food safety practice related questions were considered as having “poor level of practice.” Those who scored more than the mean value were considered as having “good level of practice” [13, 14, 36].

#### 2.7.2. Food Safety Knowledge Level

The respondents who scored less than or equal to the mean value of their responses to 10 food safety knowledge related questions were considered as having “poor level of knowledge.” Those who scored more than the mean value were considered as having “good level of knowledge” [13, 14, 36].

#### 2.7.3. Food Safety Attitude Level

The respondents who scored less than or equal to the mean value of their responses to 9 questions about their attitude towards food safety were considered as having “poor level of attitude.” Those who scored more than the mean value were considered as having “good level of attitude” [13].

## 3. Results

### 3.1. Sociodemographic Information

All the 423 study participants responded to the questionnaire, thus resulting in 100% response rate. The mean (±SD) age of the study participant was 39.84 ± 1.10 years. One hundred seventy-two (40.7%) of the respondents could not read and write. The mean monthly income of respondents was Ethiopian* birr* (ETB) 1687.9 (approximately, US dollars 62.5 at the rate of USD 1 = ETB 27). Only ninety-one (21.5%) of the study participants have attended informal food safety training during the years 2016–2018 (Table 1).

### 3.2. Food Safety Knowledge, Attitude, and Practice Levels of Mothers

**Figure 1:**
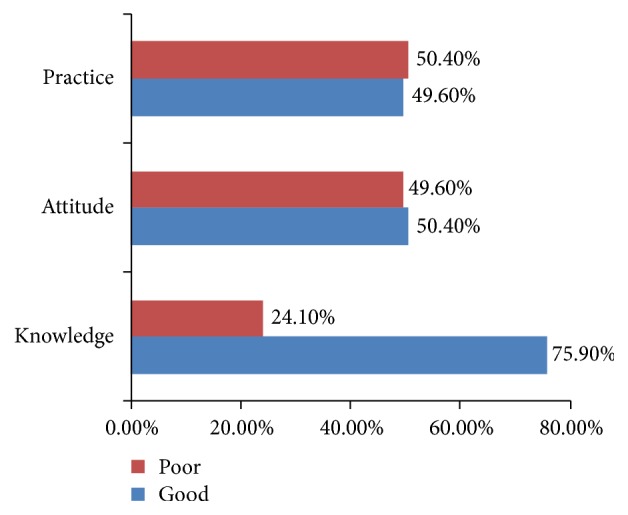
Food safety knowledge, attitude, and practice level of mothers in Debarq town, northwest Ethiopia, March – April 2018.

Of the 423 respondents, 321 (75.9%) had a good level of knowledge, and 213 (50.4%) had a good level of attitude about the food safety whereas 210 (49.6%) mothers had a good level of food safety practice and 213 (50.4%) had a poor level of self-reported practice (Figure 1).

### 3.3. Factors Associated with Food Safety Practice of Mothers

Age, marital status, income, educational status, food safety training experience, family size, knowledge, and attitude of mothers about food safety had p-values less than 0.2 in the bivariable logistic regression. Of these factors, only educational status, knowledge, and attitude were significantly associated with food safety practice in the multivariable regression analysis (Table 2).

As compared with the mothers who cannot read and write, the probability of a good level of self-reported food safety practice among mothers who had secondary educational status was 3.09 times higher [AOR 3.09, 95% CI: 1.54, 6.20] and among mothers who had college and university level educational status 2.95 times higher [AOR 2.95, 95% CI: 1.22, 7.12].

The odds of food safety practice were 2.49 times higher [AOR 2.49, 95% CI: 1.41, 4.40] among mothers who had a good level of knowledge than those who had a poor level of knowledge.

The probability of food safety practice was 3.67 times higher [AOR 3.67, 95% CI: 2.27, 5.94] among mothers who had a good level of attitude towards food safety than those who had a poor level of attitude.

## 4. Discussion

Only 49.6% of the study participants in the current study had a good level of food safety practice. This is lower than studies conducted among food vendors in north central Nigeria [37], Sri Lanka [38], and Khaza bazar, India [39], but higher than studies conducted in Arba Minch, Ethiopia [14]. This discrepancy might be because of the difference in the study period, sample size, and sociodemographic conditions of the study subjects.

The study participants have higher food safety knowledge score as compared to their attitude and practice. This study showed that 75.9% of the study subjects had a good knowledge which was higher than studies conducted among Saudi Arabia mothers [40], youth in Malaysia [41], Cassava processors in Nigeria [42], women working in Alexandria University, Egypt [43], and women of Khaza bazar, India [39], and amongst food handlers in the Republic of Ireland [44]. This difference might be because of the time of the study, different tools used to assess the level of knowledge, sample size, and socioeconomic difference among the study units.

About 50.4% of the study subjects in the current study had a good attitude. This result is lower than a study conducted in Ghana Acra [45] and India [39] but in line with a study conducted in Nigeria [42]. The difference might be because of the time of the data and method used in the current study is self-reported which might be affected by social desirability bias.

Good level of food safety practice of mothers in Debarq town was associated with good levels of educational status, knowledge, and attitude. However, only about half the number of study participant mothers (49.6%) had a good level of food safety practice and the other half (50.4%) had a poor level. Thus, the risk of occurrence of food-borne illnesses to the household members, including children with under-five years of age as they are least resistant to food-borne diseases, may be at least 50% as they are likely to be exposed to the unsafe food available because of the poor level of food safety practice of 50% of the food handling mothers at households.

The food safety practice was significantly associated with the educational status of mothers. The food safety practice was higher among study subjects with secondary school and above educational status. This result is in line with different earlier studies [40, 46–48].

Mothers with a good score of knowledge level had better food safety practice. This is in line with earlier studies [13, 16, 30, 39, 49]. Study subjects with a good score of attitude toward food safety also had reported better food safety practice. This finding is more or less similar with earlier studies [41, 50]. However, the scores for both the good and poor levels of attitude and practice remained at about 50% that is far below the score of a good level of knowledge (75.9%). Thus, considerable gap exists between the level of knowledge on the one hand and the levels of attitude and practice among mothers in Debarq. More or less similar gap in hand washing knowledge and practice prevails in other places in Ethiopia [35, 51, 52]. Not only does the disparity between the food safety related knowledge and practice exist in Ethiopia but it is a phenomenon in other developing countries as well [53–57].

Training was not associated with the food safety practice of mothers in the current study. This absence of association does not mean that training was not an important factor. However, there could be other reasons that did not make the mothers apply their training in practice. Mothers, because of the high workload, sometimes are careless to put the knowledge they have gained into practice. This might also be because food safety training is haphazardly organised and contents of basic food safety knowledge are lacking.

## 5. Conclusion

About half the number of mothers in Debarq town had a good level of self-reported food safety practice and the other half had a poor level. Overall, their food safety practice level was found low as compared with earlier studies. There was no correlation with the level of food safety practice and some demographic variables (age, marital status, income, and family size). Educational status, knowledge, and attitude were identified as the factors having stronger significant association with the food safety practice of mothers. Therefore, improving the educational status, food safety knowledge, and positive attitude of communities, particularly of mothers, through frequent public health awareness campaigns/trainings are important interventions to enhance their level of food safety practice.

Finally, this research did not include direct observations such as food and drinking water quality storage and handling food, cooking setting, and washing facility at households. Hence, the self-reported food safety practice level of mothers might be high because of social desirability bias. The tool used in this study was not a standard. In addition, because of scarce studies focusing on food safety practice of mothers at household level in both Ethiopia and other countries the comparison of results is difficult.

## Figures and Tables

**Table 1:** Sociodemographic information of study participants in Debarq town, northwest Ethiopia, March – April 2018.

Sociodemographic variables	Frequency (n)	Percent (%)
Age		
18-31 years	114	27.0
32 – 40 years	118	27.9
41-48 years	89	21.0
49-80 years	102	24.1
Educational status		
Cannot read or write	172	40.7
Primary education	96	22.7
Secondary education	84	19.9
College or University	71	16.8
Marital status		
Single	65	15.4
Married	242	57.2
Divorced	57	13.5
Widowed	59	13.9
Average monthly income		
200-800	111	26.2
801-1100	103	24.3
1101-2145	104	24.6
>2145	105	24.8
Religion		
Christian	333	78.7
Muslim	90	21.3
Ethnicity		
Amhara	391	92.4
Qimant	21	5.0
Others	11	2.6
Family size		
2-5	310	73.3
>5	113	26.7
Ever heard about food safety		
Yes	333	78.7
No	90	21.3
Ever attended food safety training		
Yes	142	33.6
No	281	66.4
Attended food safety training in the past two years (n=142)		
Yes	91	21.5
No	332	78.5

**Table 2:** Bivariable and multivariable logistic regression analysis of factors associated with food safety practice among mothers of Debarq town, northwest Ethiopia, March – April 2018.

*Variables *	*Practice *		*COR with 95*%* CI*	*AOR with 95*%* CI*
	Good	poor		
Age				
18-31	71	43	3.03(1.74,5.28)	1.20(0.56,2.60)
32-40	66	52	2.33 (1.35,4.01)	1.16(0.57,2.33)
41-48	37	52	1.30(0.73,2.34)	0.81(0.40,1.64)
49-80	36	66	1	
Marital status				
Single	41	24	2.04 (0.99, 4.20)	0.95(0.39,2.34)
Married	122	120	1.20(0.68, 2.16)	1.12(0.56,2.24)
Widowed	21	38	0.66(0.31,1.39)	0.90(0.37,2.20)
Divorced	26	31	1	
Monthly income				
200-800	50	61	1	
801-1100	47	56	1.02(0.60,1.76)	0.91(0.49,1.69)
1101-2145	50	54	1.13(0.66,1.93)	0.74(0.39,1.40)
2146-11001	63	42	1.83(1.07,3.14)	0.85(0.41,1.75)
Educational status				
Cannot read and write	51	121	1	
Primary	47	49	2.28(1.36, 3.81)	1.42(0.77,2.60)
Secondary	58	26	5.29(3.00, 9.33)	3.09(1.54,6.20)^*∗∗*^
Diploma and above	54	17	7.54(3.99, 14.23)	2.95(1.22,7.12)^*∗*^
Ever attended food safety training				
Yes	88	54	2.12(1.41, 3.21)	1.37(0.65,2.88)
No	122	159	1	
Attended food safety training the past 2 years				
Yes	53	38	1.56(0.97, 2.48)	0.75(0.33,1.69)
No	157	175	1	
Family size				
1-5	170	140	2.22(1.42, 3.46)	1.51(0.88,2.59)
6-12	40	73	1	
Knowledge				
Good	183	138	3.68(2.25, 6.03)	2.49(1.41, 4.40)^*∗∗*^
Poor	27	75	1	
Attitude				
Good	151	62	6.23(4.09, 9.50)	3.67(2.27, 5.94)^*∗∗∗*^
Poor	59	151	1	

^*∗*^Significant < 0.05; ^*∗∗*^significant at p < 0.01; ^*∗∗∗*^significant at p < 0.001.

## Data Availability

The data used to support the findings of this study are available from the corresponding author upon request.

## List of Abbreviations

AOR: Adjusted odds ratio
CI: Confidence interval
COR: Crude odds ratio
Km: Kilometre
Km^2^: Kilometre square
SD: Standard deviation
SPSS: Statistical Package for Social Sciences.
